# Porcine sapovirus replication is restricted by the type I interferon response in cell culture

**DOI:** 10.1099/vir.0.071365-0

**Published:** 2015-01

**Authors:** Myra Hosmillo, Frédéric Sorgeloos, Rintaro Hiraide, Jia Lu, Ian Goodfellow, Kyoung-Oh Cho

**Affiliations:** 1 Laboratory of Veterinary Pathology, College of Veterinary Medicine, Chonnam National University, 300 Yongbong-dong, Buk-gu, Gwangju 500-757, South Korea; 2 Division of Virology, Department of Pathology, University of Cambridge, Addenbrooke’s Hospital, Hills Road, Cambridge CB2 2QQ, UK

## Abstract

*Porcine sapovirus* (PSaV) of the family *Caliciviridae*, is the only member of the genus *Sapovirus* with cell culture and reverse genetics systems. When combined with the piglet model, these approaches provide a system to understand the molecular basis of sapovirus pathogenesis. The replication of PSaV in cell culture is, however, restricted, displaying an absolute requirement for bile acids and producing lower levels of infectious virus than other caliciviruses. The effect of bile acids has previously been linked to a reduction in the signal transducer and activator of transcription (STAT1)-mediated signalling pathway. In the current study, we observed that even in the presence of bile acids, PSaV replication in cell culture was restricted by soluble factors produced from infected cells. This effect was at least partially due to secreted IFN because treatment of cells with recombinant porcine IFN-β resulted in significantly reduced viral replication. Moreover, IFN-mediated signalling pathways (IFN, STAT1 and the 2′,5′-oligoadenylate synthetase) were activated during PSaV infection. Characterization of PSaV growth in cell lines deficient in their ability to induce or respond to IFN showed a 100–150-fold increase in infectious virus production, indicating that the primary role of bile acids was not the inactivation of the innate immune response. Furthermore, the use of IFN-deficient cell lines enabled more efficient recovery of PSaV from cDNA constructs. Overall, the highly efficient cell culture and reverse genetics system established here for PSaV highlighted the key role of the innate immune response in the restriction of PSaV infection and should greatly facilitate further molecular studies on sapovirus host–cell interactions.

## Introduction

Caliciviruses have emerged as important pathogens for both humans and animals. Within the family *Caliciviridae*, members of the genera *Norovirus* and *Sapovirus* are a significant cause of viral gastroenteritis in humans worldwide (Blanton *et al.*, 2006). Despite their importance, no efficient cell culture system to study human caliciviruses has been established (Duizer *et al.*, 2004). Due to their similarity, the study of animal caliciviruses has provided insights into the basic biology of human caliciviruses. Currently, murine norovirus (MNV) is used as a model system as it grows efficiently in immortalized macrophage cell lines and it is possible to recover infectious MNV by reverse genetics (Yunus *et al.*, 2010). However, MNV does not typically cause diarrhoea or gastroenteritis in immunocompetent mice (Wobus *et al.*, 2006). To date, porcine sapovirus (PSaV) is the only calicivirus that replicates in cell culture (Chang *et al.*, 2004), is amenable to reverse genetics (Chang *et al.*, 2005) and reproducibly causes diarrhoea in the natural immunocompetent host (Wang *et al.*, 2006).

PSaV is known to circulate in wide geographical regions including America, Europe and Asia. In the USA, PSaV has been detected in at least 62 % of pigs tested (Wang *et al.*, 2006). This frequency is reported to be lower in Europe with a prevalence of 11.1 %, suggesting an endemic persistence of the virus across European pig farms (Reuter *et al.*, 2010). In Asian countries such as China, Taiwan, South Korea and Japan, PSaV shows a prevalence of between 0.6 and 40 % of the pigs examined (Jeong *et al.*, 2007; Nakamura *et al.*, 2010; Wang *et al.*, 2006; Yu *et al.*, 2012). This widespread distribution indicates the importance of swine as potential sapovirus reservoirs (Thiry *et al.*, 2012). Whilst PSaVs have limited health implications for swine, previous reports have raised public health concerns as PSaV is genetically highly related to human sapovirus and can act as an animal reservoir for viral recombination (Martella *et al.*, 2008). Despite this potential threat, there is limited understanding of the mechanisms of virus replication.

To study the molecular basis of PSaV replication and pathogenesis, the PSaV Cowden strain has been adapted to grow in a pig kidney continuous cell line (LLC-PK), but requires the presence of bile acids, specifically sodium glycochenodeoxycholate (GCDCA) (Chang *et al.*, 2004). A reverse genetics system has also been developed, enabling virus recovery from *in vitro* transcribed capped PSaV RNA (Chang *et al.*, 2005). However, when compared with the other caliciviruses for which culture systems have been established, PSaV replication is reduced, typically producing 100–1000-fold lower titres of infectious virus than MNV (Chaudhry *et al.*, 2007).

Viruses subdue the host restriction and innate immune responses to infection using various approaches (Grinde, 2013; Oldstone, 2007). In PSaV, bile acids were identified as an essential cofactors enabling virus infection in cell culture (Chang *et al.*, 2004). Whilst the main function of bile acids in PSaV infection is unclear, previous studies suggested a role in supporting the downregulation of IFN signalling by reducing phosphorylation of the signal transducer and activator of transcription (STAT1) (Chang *et al.*, 2004). A number of slow-growing, vaccine and WT viruses have been propagated in IFN-deficient cell lines (Young *et al.*, 2003). A general approach to produce IFN-deficient cell lines has been achieved by ectopically expressing viral proteins known to antagonize the IFN pathway, such as NPro of bovine viral diarrhoea virus (BVDV) or the V protein of parainfluenza virus type 5 (PIV5; formerly known as simian virus type 5) (Randall *et al.*, 2012; Sherwood *et al.*, 2007; Young *et al.*, 2003). NPro is a protein product of persistent strains of BVDV and functions by preventing IFN regulatory factor 3 (IRF-3) binding to DNA as well as targeting IRF-3 for polyubiquitination and degradation by the proteasome (Hilton *et al.*, 2006; Seago *et al.*, 2007). The PIV5 V protein binds to MDA5 (melanoma differentiation associated protein 5) preventing its binding to dsRNA and associated downstream signalling (Childs *et al.*, 2007). In addition, it blocks IFN signalling by targeting STAT1 for proteasome-mediated degradation (Killip *et al.*, 2011) and interacts with RNA helicase LGP2 targeting RIG-I (retinoic acid inducible gene I)-dependent IFN induction (Childs *et al.*, 2007).

Based on the growing body of literature on the role of innate immune responses in controlling calicivirus replication in cell culture, we examined the sensitivity of PSaV to the type I IFN response as well as the ability of BVDV NPro or PIV5 V proteins to improve the replication of PSaV. Unexpectedly, we observed that the requirement for GCDCA during PSaV infection was independent of the innate immune response.

## Results

### Constitutive expression of BVDV NPro and PIV5 V in porcine LLC-PK cells inhibits the PSaV-mediated IFN-mediated signalling pathway

**Fig. 1.**
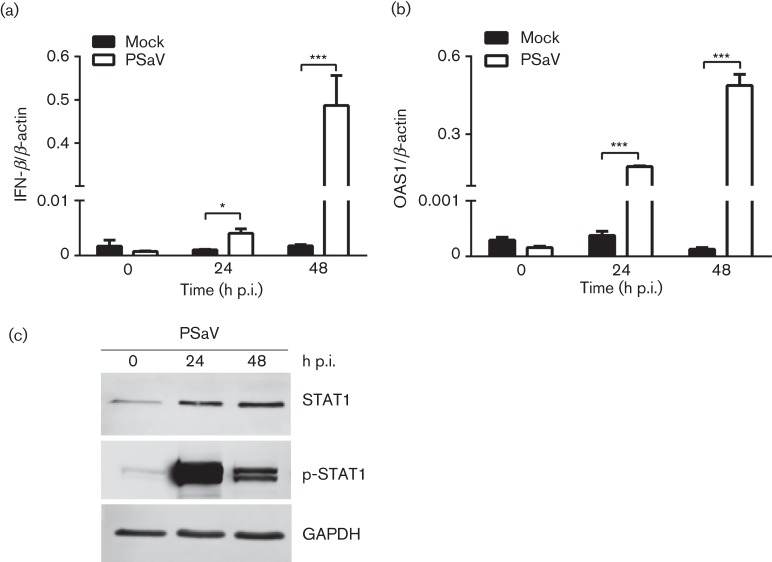
PSaV infection triggers innate immune responses. (a, b) LLC-PK cells were infected with PSaV at m.o.i. 0.2 TCID_50_ per cell, and gene expression levels of IFN-β (a) and OAS1 (b) were analysed by quantitative real-time (qRT)-PCR at 0, 24 and 48 h post-infection (p.i.). All experiments were performed three independent times and results are expressed as mean±sd from triplicate samples. Statistically significant values: *P<0.05, **P<0.005 and ***P<0.0001. (c) PSaV-infected LLC-PK cells were lysed at 0, 24 and 48 p.i., and the protein expression of STAT1, p-STAT1 and GAPDH was determined by Western blotting.

To examine the effect of PSaV replication on the stimulation of the type I IFN pathway, we first examined the mRNA levels of porcine IFN-β and the 2′,5′-oligoadenylate synthetase (OAS1), and the protein levels of phosphorylated (p-STAT1) and total STAT1 during PSaV infection in the currently used LLC-PK porcine kidney cell line. As shown in Fig. 1(a, b), IFN-β and OAS1 mRNAs increased over the course of PSaV infection, resulting in a 260-fold increase in IFN-β and a 2800-fold stimulation of OAS1 mRNA levels at 48 h post-infection (p.i.). To determine whether increased OAS1 gene expression was correlated with the induction of STAT1 or p-STAT1, protein levels of STAT1 and p-STAT1 were examined by Western blotting. As expected, STAT1 and p-STAT1 protein levels were increased at 24 and 48 h p.i., but not the expression level of glyceraldehyde 3-phosphate dehydrogenase (GAPDH) used as a control. This increase was correlated with the transient phosphorylation of STAT1 observed at 24 and 48 h p.i. (Fig. 1c). These results indicated that PSaV readily stimulated the IFN pathway and this observation led us to hypothesize that the innate immune responses might be responsible for the slow establishment of PSaV infection in cell culture.

To test this hypothesis, we used lentiviral vectors to generate LLC-PK cells constitutively expressing either BVDV NPro or PIV5 V proteins, two well-characterized innate immune antagonists. In brief, the BVDV NPro protein was from a non-cytopathic, persistent biotype of BVDV which could effectively block IFN production by degrading IRF3, thereby preventing the activation of the innate immune system (Peterhans & Schweizer, 2013). The PIV5 V protein, however, targeted IFN production as well as antiviral signalling by targeting STAT1, MDA5 and LGP2 for proteasomal degradation (Andrus *et al.*, 2011; Childs *et al.*, 2007; Randall *et al.*, 2012).

**Fig. 2.**
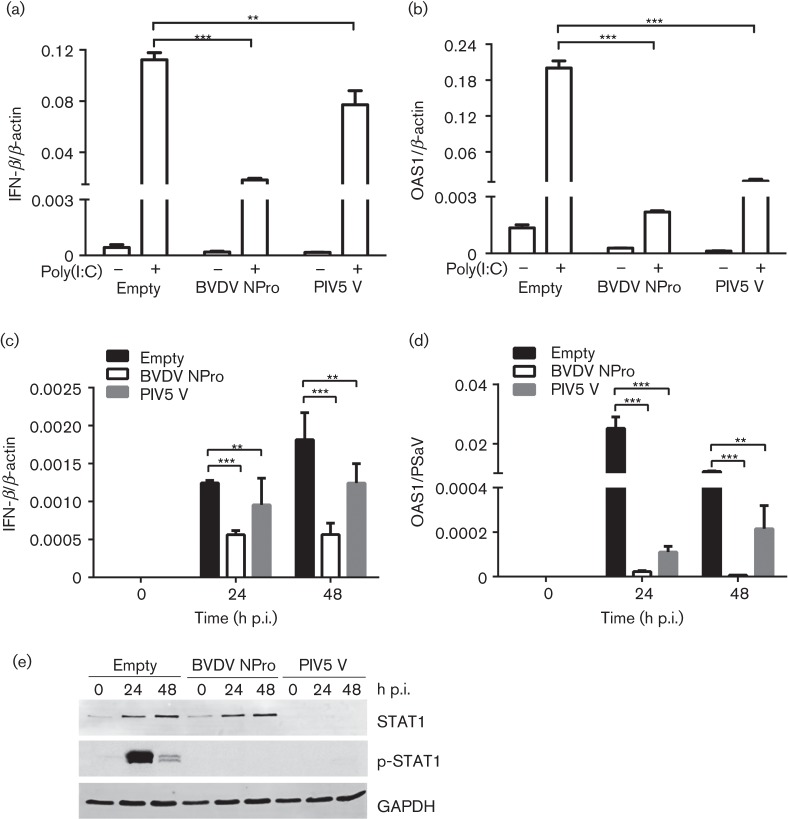
Transduction of BVDV NPro and PIV5 V inhibits IFN production and signalling in porcine LLC-PK cells. (a, b) LLC-PK cells transduced with the indicated constructs were transfected with poly I : C, and levels of IFN-β (a) and OAS1 (b) mRNA levels were analysed by qRT-PCR at 7 h p.t. All experiments were performed three independent times and results are expressed as mean±sd from triplicate samples. Statistically significant values: *P<0.05, **P<0.005 and ***P<0.0001. (c, d) LLC-PK cells expressing the indicated constructs were infected with PSaV at m.o.i. 0.2 TCID_50_ per cell, and expression levels of IFN-β (c) and OAS1 (d) mRNAs and the PSaV protease gene were analysed by qRT-PCR at 0, 24 and 48 h p.i., and then the ratio between the expression levels of IFN-β or OAS1 mRNAs and the PSaV protease gene were graphed. All experiments were performed three independent times and results are expressed as mean±sd from triplicate samples. Statistically significant values: *P<0.05, **P<0.005 and ***P<0.0001. (e) LLC-PK cells expressing the indicated constructs were infected with PSaV, and lysed at 0, 24 and 48 h p.i. Expression levels of STAT1, p-STAT1 and GAPDH were determined by Western blotting.

To verify the ability of NPro and V proteins to inhibit IFN-β induction and signalling in porcine LLC-PK cells, transduced LLC-PK cells were transfected with polyinosinic acid : polycytidylic acid [poly(I : C)], and the levels of IFN-β and OAS1 mRNAs were quantified by quantitative real-time (qRT)-PCR. The cellular response to poly(I : C) transfection was observed 4 h post-transfection (p.t.), suggesting that LLC-PK cells were IFN competent. IFN-β and OAS1 mRNA levels were elevated in control cells transduced with the empty vector as expected (Fig. 2a, b). In striking contrast, cells expressing BVDV NPro showed significantly reduced IFN-β and OAS1 induction when compared with the levels observed in control cells (Fig. 2a, b). In the case of PIV5 V-expressing cells, whilst IFN-β mRNA was moderately upregulated, OAS1 gene expression was readily inhibited in response to poly(I : C) transfection (Fig. 2a, b). These data indicated that BVDV NPro and PIV5 V proteins could efficiently block IFN induction and signalling in LLC-PK cells, despite them being of porcine origin.

The effect of PSaV on the innate immune response in transduced LLC-PK cells was examined following virus infection (Fig. 2c, d). As expected, IFN-β mRNA levels were increased in a time-dependent manner during the course of infection, but were significantly reduced in cells expressing NPro or V protein (Fig. 2c). Similarly, cells expressing NPro or V protein showed significantly reduced levels of OAS1 mRNA induction (Fig. 2d). To test whether PSaV-stimulated IFN-β and OAS1 genes were correlated with upregulation of STAT1 and p-STAT1, the expression levels of STAT1 and p-STAT1 were examined by Western blotting. As shown in Fig. 2(e), STAT1 and p-STAT1 protein levels were enhanced by 24 and 48 h p.i. in control and BVDV NPro-expressing cells, but not the expression level of GAPDH (Fig. 2e). As expected, however, no p-STAT1 or non-phosphorylated STAT1 was detected in PIV5 V-expressing cells, which is known to target STAT1 for proteasomal degradation (Fig. 2e).

Together, these results showed that PSaV was efficiently sensed by LLC-PK cells during infection, and that the NPro and V proteins were active in cells from porcine origin.

### Restriction of PSaV growth is due to production of soluble factors

**Fig. 3.**
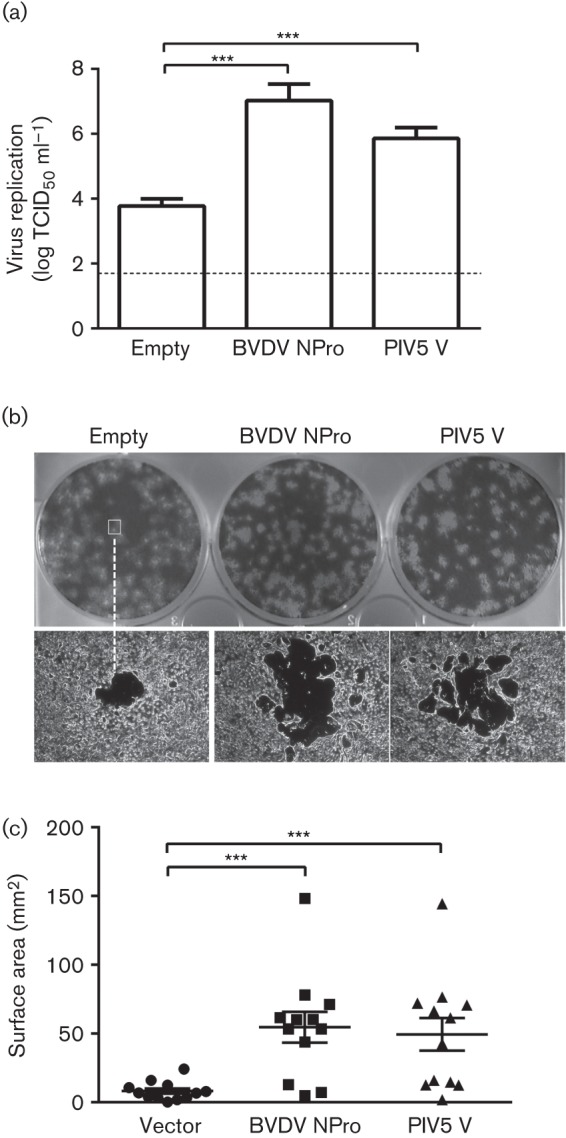
PSaV infection is restricted by soluble factors. (a) Either empty vector-, BVDV NPro- or PIV5 V-transduced cell lines were infected with PSaV at m.o.i. 0.2 TCID_50_ per cell. Cells were harvested at 48 h p.i., and frozen and thawed twice. Clarified virus supernatants were subjected to UV exposure and used to pretreat parental LLC-PK cells for 16 h. Cells were then infected and viral replication was quantified by TCID_50_. It is important to note that virus stock used for inoculation was prepared from NPro-expressing cells. (b) Plaque phenotypes of PSaV were examined in monolayers of transduced cells infected with PSaV at 1 or 10 p.f.u. per plate. Representative plaques for each cell line are shown. (c) Plaque sizes were measured and analysed by ImageJ v1.44i software. All experiments were performed three independent times and results are expressed as mean±sd from triplicate samples. Statistically significant values: *P<0.05, **P<0.005 and ***P<0.0001. The dashed line was used to indicate the limit of detection by TCID_50_.

To determine if soluble factors could account for the slow growth of PSaV in cell culture, LLC-PK cells transduced with BVDV NPro, PIV5 V or empty vector were infected with PSaV and viral supernatants were collected. The supernatants were then UV-treated to inactivate any virus present. These were subsequently used to pretreat parental LLC-PK cells and the effect on virus replication was examined. As a control, incubation of cells with UV-treated supernatants did not lead to the production of detectable levels of infectious virus and confirmed the efficacy of UV treatment (data not shown). Contrastingly, preincubation of LLC-PK cells with supernatants originating from control cells resulted in a virus yield 100–1000-fold lower than that obtained from cells preincubated with supernatants from BVDV NPro- or PIV5 V-expressing cell lines (Fig. 3a). It is worth noting that the virus inoculum used to initiate infections was prepared in BVDV NPro-expressing cells to minimize any soluble inhibitory factors that might have been present in the virus inoculum.

To further characterize the role of these soluble inhibitory factors, we compared the plaque morphology of PSaV in the three cell lines. Individual plaques in cells transduced with the empty vector were small and surrounded by a ring of opaque cells (Fig. 3b). The cells surrounding the region of cytopathic effect (CPE) were likely to reflect the limitation of PSaV cell-to-cell spread, thus only small-sized plaques were formed. Plaque sizes from cells transduced with BVDV NPro and PIV5 V proteins were six to seven times larger in surface area than the plaques observed in cells transduced with the empty vector (Fig. 3c). The observed increase in plaque size was consistent with the ability of virus to spread in the absence of a functional IFN pathway.

### Porcine IFN-β protects cells during PSaV infection

**Fig. 4.**
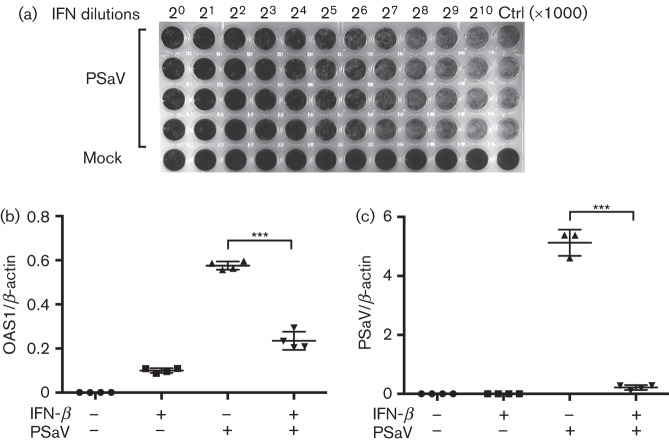
Porcine IFN-β protects cells from PSaV infection. (a) LLC-PK cells were treated with either control (Ctrl) or porcine recombinant IFN-β in a twofold dilution series and incubated for 16 h prior to PSaV infection. Plates were stained with methylene blue after 48 h and cells were observed for CPE. (b, c) A similar procedure of control and porcine IFN treatment was performed in 24-well plates before and during PSaV infection. PSaV was infected at m.o.i. 0.1 TCID_50_ per cell, unadsorbed viruses were washed after 3 h, and OAS1 (b) and PSaV (c) mRNA levels were quantified by qRT-PCR. All experiments were performed three independent times and results are expressed as mean±sd from triplicate samples. Statistically significant values: *P<0.05, **P<0.005 and ***P<0.0001.

The results described above demonstrated that IFN-mediated signalling occurred during PSaV infection (Figs 1 and 2) and that soluble factors secreted from infected cells inhibited PSaV replication (Fig. 3). To evaluate whether IFN-β was a major component of the soluble inhibitory factors secreted during PSaV infection, parental LLC-PK and transduced cell lines were treated with recombinant porcine IFN-β produced by transient expression of the porcine IFN-β gene in human 293T cells. Protection from PSaV-induced CPE was observed down as far as a 1/64000 (1000×2^6^) dilution of the IFN-β-containing supernatant (Fig. 4a). No protection from CPE was observed in cells incubated with control supernatant (Fig. 4a).

To quantify the protection provided by IFN-β against PSaV infection, qRT-PCR analysis was performed following IFN-β treatment and virus infection. IFN-β treatment resulted in a 120-fold increase in OAS1 mRNA when compared with mock-treated samples. In PSaV-infected cells, a 620-fold increase in OAS1 mRNA was observed that was reduced to 270-fold when cells were pretreated with IFN-β (Fig. 4b). This decreased effect correlated with a >23-fold reduction in viral RNA production, confirming the IFN sensitivity of PSaV (Fig. 4c). Taken together, an IFN-mediated signalling pathway was evoked during PSaV infection in the cells.

### Bile acid requirement for PSaV growth is independent of the IFN response

**Fig. 5.**
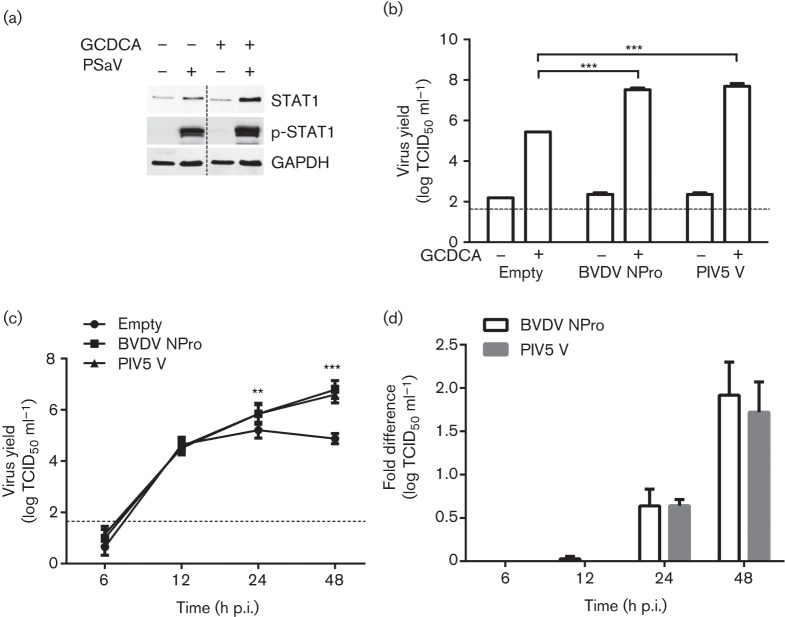
PSaV virus yield is improved in IFN-deficient cell lines, but independent of any GCDCA requirement. (a) PSaV was inoculated into LLC-PK cells in the absence or presence of GCDCA. Cells were lysed at 24 h p.i., and protein expression levels of STAT1, p-STAT1 and GAPDH were examined by Western blotting. (b) Subsequently, an infection assay was performed in either empty vector-, BVDV NPro- or PIV5 V-transduced cell lines. Cells were infected at m.o.i. 0.2 TCID_50_ per cell in the absence or presence of GCDCA (200 µM) and virus yield was measured at 48 h p.i. by TCID_50_. (c) Multistep growth kinetics of PSaV was characterized in the three cell lines at 6, 12, 24 and 48 h p.i. All experiments were performed three independent times and results are expressed as mean±sd from triplicate samples. Statistically significant values: *P<0.05, **P<0.005 and ***P<0.0001. The dashed line was used to indicate the limit of detection by TCID_50_.

The replication of PSaV Cowden strain in LLC-PK cells is absolutely dependent on the presence of GCDCA – a conjugated bile acid (Chang *et al.*, 2004). Previous studies suggested that the requirement for GCDCA may be dependent on the initial cAMP and STAT1 signalling pathways, modulating host gene expression that resulted in enhanced PSaV replication (Chang *et al.*, 2002, 2004). However, the exact mechanism for the requirement for GCDCA remains unknown. To examine the effect of GCDCA on STAT1 and p-STAT1 during PSaV infection of naive LLC-PK cells, the expression levels of total STAT1 and p-STAT1 were analysed in the presence and absence of GCDCA with PSaV infection. The levels of STAT and p-STAT1 were enhanced upon sapovirus infection irrespective of GCDCA addition (Fig. 5a). To further investigate the role of GCDCA in relation to the IFN response, LLC-PK cells expressing either BVDV NPro, PIV5 V or transduced with the empty vector were infected with PSaV in the absence or presence of GCDCA. Bile acids dramatically increased PSaV titres in all cell lines analysed (Fig. 5b). These data suggested that the requirement of PSaV for GCDCA in cell culture is independent of IFN pathway inhibition and could be related to other cellular pathways.

To characterize the replication kinetics of PSaV in IFN-deficient cells, a multi-cycle growth curve analysis was performed in the transduced cell lines in the presence of GCDCA (Fig. 5c). We observed a rapid increase in virus titre from 6 to 24 h p.i. in all of the cell lines; however, in the cell line transduced with the empty vector, virus replication plateaued at 24 h p.i. In striking contrast, virus titres obtained from BVDV NPro- and PIV5 V-expressing cells continuously increased over time up to 48 h p.i., resulting in a final titre ~100-fold higher in cells expressing BVDV NPro or PIV5 V proteins (Fig. 5c, d). These data clearly showed that in cell lines where the innate immune response was attenuated, viral replication was greatly enhanced, enabling multiple cycles of reinfection.

### Inactivation of the IFN response in porcine cells enhances the ability to recover PSaV by reverse genetics

**Fig. 6.**
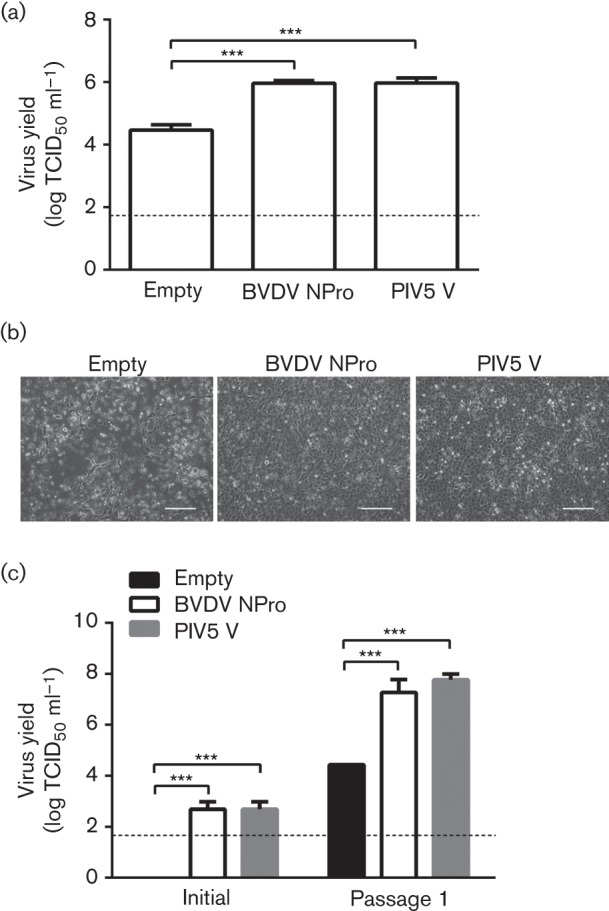
PSaV reverse genetics generates better infectious yield in IFN-deficient cell lines. (a) VPg-linked PSaV RNA extracted from parental LLC-PK cells was transfected into IFN-competent and -deficient cell lines. Virus yield was quantified for each cell line by TCID_50_. (b) Capped *in vitro* transcribed PSaV RNA was transfected to the same cell lines and observation of CPE-like reactions was evident after 20 h p.t. in the vector-containing cells. Bar, 10 µm. (c) Capped *in vitro* transcribed PSaV was transfected into IFN-competent and -deficient cell lines. Cells were harvested at 4 days p.t. and recovered infectious virus was titrated by TCID_50_. All experiments were performed three independent times and results are expressed as mean±sd from triplicate samples. Statistically significant values: *P<0.05, **P<0.005 and ***P<0.0001. The dashed line was used to indicate the limit of detection by TCID_50_.

The observed increased replication in PSaV growth in IFN-deficient cell lines enabled the production of high-titre virus stocks (>10^7^ infectious units ml^−1^) and may, therefore, allow the replication of debilitated viruses obtained by reverse genetics. Indeed, virus recovery by reverse genetics is intrinsically limited by cellular responses that often result in suboptimal virus production and recovery (Yunus *et al.*, 2010). To investigate whether BVDV NPro- and PIV5 V-expressing cells allowed more efficient recovery of infectious PSaV by reverse genetics, we initially examined the virus yield following transfection of VPg-linked viral RNA isolated from infected cells (Fig. 6a). The yield of infectious virus recovered from BVDV NPro- and PIV5 V-expressing cells was typically ~13-fold higher than the yield obtained from control cells (Fig. 6a). The ability to rescue recombinant PSaV from *in vitro* transcribed capped RNA produced from a PSaV cDNA clone was also improved (Fig. 6c). Interestingly, we observed that the presence of either BVDV NPro or PIV5 V protein significantly reduced the toxicity of RNA transfection in LLC-PK cells. We observed significant levels of CPE 15 h p.t. of capped RNA in cells containing the vector alone, whereas BVDV NPro- or PIV5 V-transduced cells displayed a normal morphology (Fig. 6b). As reported previously, transfection of LLC-PK cells with RNA resulted in the rapid appearance of toxicity that was not linked directly to viral replication (Nguyen *et al.*, 2014). Titration of virus recovered from the initial transfection indicated that the observed CPE was not reflective of virus replication. Given that this apparent transfection-induced toxicity was not observed in either BVDV NPro- or PIV5 V-expressing cell lines (Fig. 5b), it is likely to have been a reflection of the activation of the innate immune response leading to apoptosis and cell death (Apelbaum *et al.*, 2013; Bekisz *et al.*, 2010). The yield of recombinant virus recovered either after the initial transfection or subsequent passage in the same cell type was significantly enhanced in either BVDV NPro- or PIV5 V-expressing lines, highlighting the utility of these lines for the improved recovery of recombinant PSaV in cell culture.

## Discussion

PSaV is a prototype enteric sapovirus and is closely related to human sapoviruses that, together with human noroviruses, are well established as major causative agents of gastroenteritis outbreaks worldwide in humans (Blanton *et al.*, 2006; Chang *et al.*, 2005; Glass, 2013). PSaV remains the only cultivable member of the genus *Sapovirus* and represents therefore a useful model to understand sapovirus pathogenesis (Chang *et al.*, 2002, 2004). However, molecular studies on PSaV remain difficult due to slow virus replication and the low viral yield obtained from reverse genetics. Therefore, we aimed to examine the mechanism behind the reduced viral growth in cell culture to enable us to better understand the molecular mechanisms of PSaV replication and pathogenesis.

Although several factors may contribute to the low virus yield in immortalized cells, studies on other caliciviruses have indicated that one of the most important factors is the sensitivity to the host innate immune response (Changotra *et al.*, 2009; McFadden *et al.*, 2011). Thus, the control of IFN or other related restrictive factors becomes the critical determinant in obtaining higher titres of PSaV and more efficient virus recovery. Recent studies have demonstrated the critical effect of type I IFN on MNV-1 viral production, and it was demonstrated that a STAT1-dependent IFN pathway plays a key role in controlling MNV replication both *in vitro* and *in vivo* (Changotra *et al.*, 2009; Karst, 2011). Many viruses have evolved diverse strategies to avoid or antagonize the innate immune response, yet there are a number of viruses that still have unknown or limited evasion mechanisms. In most cases, however, these viruses are particularly slow-growing or attenuated strains. Notably, viruses with this characteristic, including respiratory syncytial virus, bunyaviruses and human enteric adenovirus, are known to grow better in IFN-deficient cell lines (Sherwood *et al.*, 2007; Young *et al.*, 2003).

To date, none of the characterized sapovirus proteins are known to antagonize the IFN response. Our observation that IFN-β and OAS1 mRNA levels are significantly increased during PSaV infection in LLC-PK cells led us to hypothesize that PSaV is restricted by the IFN-β-dependent innate immune response in cells. Our data are consistent with this hypothesis as we observed improved PSaV replication using cell lines deficient in IFN production or signalling (Figs 2 and 5). Following on from these observations, we were able to use these novel cell lines to provide a more robust cell culture system for PSaV and to improve the current reverse genetics system (Figs 3–6).

Incorporation of BVDV NPro or PIV5 V proteins was chosen because they are known viral gene products that have the ability to evade the innate immune system, and have shown improvement of virus growth either in bovine and human cells (Hayman *et al.*, 2007; Pérez-Cidoncha *et al.*, 2014; Young *et al.*, 2003). In particular, constructed human A549 cells constitutively expressing BVDV NPro and PIV5 V proteins enhanced replication of avian viruses compared with unmodified cells (Hayman *et al.*, 2007). Considering these previous reports, the NPro and V proteins were used to generate pig cells deficient in their ability to induce the type I IFN response. Strikingly, these cell lines showed a 100–150-fold increase in PSaV replication (Fig. 5). To our knowledge, this is the first report showing the generation of a IFN-deficient cell line in a porcine system and this could be a useful strategy for other pig viruses as a way to curb the innate immunity restricting virus growth in cell culture.

Previous studies and the findings by the current study demonstrate that transfection of LLC-PK cells with DNA or RNA rapidly results in cell death. Whilst not confirmed in the current study, given that BVDV NPro- or PIV5 V-expressing cells did not show the same response to transfection of viral RNA, we would predict that the effect is due to activation of the innate immune response leading to apoptosis, as described previously (Chattopadhyay *et al.*, 2010). The differences in viral titres seen in Fig. 5(c) at 48 h also agree with this observation. The abrogation of the innate immune response in LLC-PK cells provides a robust system for the study of PSaV replication and an improved method to recover mutated PSaV isolates from cDNA-containing constructs. The generated IFN-deficient cell lines may underscore future studies on the significance and roles of viral and cellular proteins during PSaV replication in the host cell.

Although the addition of bile acids including the GCDCA is an essential cofactor for PSaV replication in cell culture, the mechanisms of action of bile acids have yet to be fully elucidated. Previous observations suggested that at least part of the effect of bile acids is to suppress the IFN response by decreasing p-STAT1 levels (Chang & George, 2007; Chhatwal *et al.*, 2012). Our data showed that even in the presence of GCDCA, replicating PSaV stimulated the IFN-mediated pathway, leading to induction of IFN-β, OAS1, STAT1 and p-STAT1 gene expression (Fig. 1). Moreover, PSaV growth in BVDV NPro- and PIV5 V-expressing cells remains dependent on the presence of GCDCA (Fig. 5). These data suggest that the function of GCDCA is unrelated to the IFN immune response or that it may regulate an as yet unknown aspect of the innate immune response that is unaffected by the expression of BVDV NPro or PIV5 V protein. It is important to note that PIV5 V protein degrades STAT1 and, despite this, GCDCA is still required to allow PSaV growth. Therefore, we can conclude that the previously reported effect of GCDCA on STAT1 phosphorylation is not the mechanism by which bile acids facilitate PSaV replication. This hypothesis is in agreement with a study demonstrating a role for bile acids in PSaV entry, particularly facilitating the viral release from late endosomes into the cytoplasm (Shivanna *et al.*, 2014). Bile acids have been shown to play other roles in the life cycle of important viruses. In the case of hepatitis C virus, it has been suggested that bile acids facilitate membrane alterations that could promote RNA replication, recruitment of essential cellular cofactors or activation of viral factors (Chhatwal *et al.*, 2012).

Overall, we demonstrated that the IFN response is activated during PSaV infection in the presence of bile acids, resulting in slow growth of PSaV. The use of innate immune-deficient cells successfully facilitated the efficient replication of PSaV in cell culture, leading to improved recovery of PSaV by reverse genetics. The establishment of a more efficient cell culture system for PSaV here should greatly assist in the advancement of molecular studies on host–cell interactions and life cycle, clinical diagnosis, and future vaccine development for sapoviruses.

## Methods

### 

#### Cell lines and virus.

Parental LLC-PK cells and transduced cells were grown in Eagle’s minimal essential medium (EMEM) supplemented with 10 % (v/v) FCS, 100 U penicillin ml^−1^ and 100 µg streptomycin ml^−1^ at 37 °C with 5 % CO_2_. Infectious particles of a tissue-culture-adapted PSaV Cowden strain were recovered from full-length infectious cDNA clone pCV4A (a kind gift from Dr K. O. Chang, Kansas State University, Manhattan, KS, USA) and propagated in LLC-PK cells supplemented with 200 µM GCDCA (Chang *et al.*, 2005).

#### Lentivirus vector particle production and transduction.

Vesicular stomatitis virus G-protein-pseudotyped lentiviral particles were generated by transient transfection of 293T cells grown in 100 mm culture plates using 6 µg lentiviral vector, 6 µg psPAX2 and 3 µg pMD2.G. Parental LLC-PK cells were then transduced with lentiviral supernatants and incubated for 48 h. Transduced LLC-PK cells were selected on the basis of their resistance to puromycin at a concentration of 2 µg ml^−1^. 

#### PSaV infection.

Parental or lentivirus-transduced LLC-PK cells were infected with PSaV at m.o.i. 0.2 TCID_50_ per cell in 250 µl for 3 h at 37 °C with intermittent shaking every 15 min for the first hour. Unabsorbed inoculum was then removed and cells were washed with EMEM in the presence or absence of GCDCA at 37 °C. Supernatants were harvested at 0, 6, 12, 24 and 48 h p.i., depending on the experimental setup, and titrated by TCID_50_.

#### Virus inactivation from PSaV-infected cells.

Lentivirus-transduced LLC-PK cells containing either empty, BVDV NPro or PIV5 V vectors were infected with PSaV as described above. Viral supernatants were harvested at 48 h p.i., frozen and thawed twice, and clarified by centrifugation at 13 400 ***g*** for 1 min. Each supernatant was then placed separately in 24-well plates to a fluid depth of 10 mm and exposed to 4000 mJ from a UV source for 12 min at 4 °C. Loss of viral infectivity due to UV exposure was confirmed by titration of inactivated virus preparations by TCID_50_. Inactivated virus supernatants were incubated back to parental LLC-PK cells for 16 h at 37 °C. Incubated cells were washed and inoculated with PSaV (m.o.i. 0.2 TCID_50_ per cell) as described above. Viruses were harvested at 48 h p.i. and titrations in different cell lines were performed using TCID_50_.

#### qRT-PCR analysis.

Total cellular RNA was extracted using a GenElute Mammalian Total RNA Miniprep kit (Sigma) and 100 ng was subsequently reverse transcribed using random hexamers. Primers were designed to amplify fragments of ~200 bp of IFN-β, OAS1, β-actin and PSaV, and the β-actin gene was used as an internal reference gene. Primer sequences were: IFN-β, 5′-GGAGCAGCAATTTGGCATGT-3′ (forward) and 5′-TGACGGTTTCATTCCAGCCA-3′ (reverse); OAS1, 5′-GATGGAGCTGAGGCATACCC-3′ (forward) and 5′-GGAGCCACCCTTCACAACTT-3′ (reverse); β-actin, 5′-TCTACACCGCTACCAGTTCG-3′ (forward) and 5′-GCTCGATGGGGTACTTGAGG-3′ (reverse); and PSaV, 5′-CAACAATGGCACAACAACG-3′ (forward) and 5′-ACAAGCTTCTTCACCCCACA-3′ (reverse). Standard curves were generated for all the genes measured. The values of mRNA were expressed as the quantity of the gene of interest relative to the quantity of the reference gene to obtain normalized expression values. Each sample was performed in triplicate on the same qRT-PCR plate in two independent experiments. Additional non-template and non-reverse transcriptase samples were analysed routinely as negative controls. Data were collected using a ViiA 7 Real-Time PCR System (Applied Biosystems).

#### TCID_50_ assay.

Ten-fold serial dilutions of clarified virus supernatants were prepared in EMEM supplemented with 200 µM GCDCA. Of these dilutions, 200 µl was inoculated to monolayers of parental LLC-PK cells grown on 96-well plates and incubated at 37 °C in a 5 % CO_2_ incubator. Virus titres were collected at 6 days p.i. and expressed as TCID_50_ ml^−1^ values by the Reed–Muench method (Reed & Muench, 1938).

#### Plaque phenotype analysis.

Briefly, 800 µl diluted virus stock or media alone was inoculated on LLC-PK monolayers (37 °C, 3 h) and gently shaken for the first hour every 15 min to allow virus adsorption. Cell monolayers were then washed and overlaid with 1.3 % (w/v) Avicel-containing EMEM supplemented with 2.5 % (v/v) FBS, 0.225 % (v/v) sodium bicarbonate and penicillin/streptomycin. Plates were incubated at 37 °C for 4 days. After incubation, the Avicel mixture was removed, and cells were fixed and stained with 1.6 % (w/v) methylene blue and 10 % (v/v) formaldehyde solution in 1× PBS for 30 min. Plates were washed with distilled water until the desired staining intensity was achieved. Plaque sizes were measured by capturing images using an inverted fluorescence microscopy (×4 magnification) using QCapture Pro version 5.0.1.26 (QImaging). Resulting images were analysed using ImageJ v1.44i software to the measure surface area (mm^2^) of individual plaques.

#### Virus rescue from VPg-linked PSaV RNA or capped *in vitro* transcribed PSaV cDNA clones.

LLC-PK cells containing either BVDV NPro, PIV5 V or empty vectors were seeded at a density of 1.2×10^5^ cells per well of a 24-well plate and transfected with 0.2 µg of either mock, VPg-linked PSaV RNA or capped *in vitro* transcribed PSaV RNA using 0.8 µl Lipofectamine 2000 (Invitrogen). VPg-linked PSaV RNA was prepared from total RNA extracts from PSaV-infected cells. Capped *in vitro* transcripts were derived from the full-length PSaV cDNA clone pCV4A. Transfections were carried out for 4 h, and media was replaced with EMEM containing 2.5 % (v/v) FCS and 200 µM GCDCA. At 48 h p.t., cells were harvested, frozen and thawed twice, and then titrated by TCID_50_. Supernatants harvested from transfection of capped RNA transcripts were further inoculated to freshly seeded cells and harvested after the appearance of CPE. Plates were again frozen and thawed twice, and virus recovery was evaluated by TCID_50_.

#### Recombinant porcine IFN-β production.

A DNA fragment encoding porcine IFN-β was amplified by PCR using cDNA originated from LLC-PK. The forward primer, 5′-AAAAGGATCCG**CCACC**ATGGCTAACAAGTGCATCCTCC-3′, and reverse primer, 5′-AAAATCTAGATTAGTTCCGGAGGTAATCTGTAAG-3′, were used. The *Bam*HI restriction site (underlined) and Kozak sequence (bold) are indicated in the forward primer sequence, and the *Xba*I restriction site (underlined) is indicated in the reverse primer sequence. The PCR fragment was then cloned into pcDNA3.1 using *Bam*HI and *Xba*I restriction sites. All amplified regions were verified by Sanger sequencing. The resulting pcDNA3.1 plasmid encoding the porcine IFN-β was then transfected in 293T cells using Lipofectamine 2000. Cell supernatant containing porcine IFN-β was harvested 24 h p.t. and used as IFN-β. Control IFN was obtained from supernatants collected from cells transfected with pcDNA3.1.

#### IFN-β protection assay.

Control or IFN-β supernatants were prepared at 1 : 100 dilution of the original stock and serial twofold dilutions were prepared in 96-well plates. LLC-PK cells were incubated with serial dilutions for 16 h and infected with PSaV using 400 TCID_50_. Control or IFN-β dilutions were left on the cells until harvest at 48 h p.i. Cells were fixed and stained with methylene blue as described earlier for plaque phenotype analysis. LLC-PK cells were seeded in 24-well plates and treated with 1/1000 dilution of IFN-β. PSaV were infected at m.o.i. 3.0 TCID_50_ for 3 h at 37 °C with shaking for the first hour every 15 min to allow virus adsorption. Unadsorbed viruses were washed and viral RNA was extracted at 24 h p.i. for qRT-PCR analysis.

#### Statistical analysis and software.

Statistical analyses were performed on triplicate experiments using the two-tailed Student *t*-test (Prism 6 version 6.04). Figures were generated using Adobe Photoshop CS3 and Prism 6 version 6.04.
